# CryoTEN: efficiently enhancing cryo-EM density maps using transformers

**DOI:** 10.1093/bioinformatics/btaf092

**Published:** 2025-02-27

**Authors:** Joel Selvaraj, Liguo Wang, Jianlin Cheng

**Affiliations:** Department of Electrical Engineering and Computer Science, University of Missouri, Columbia, MO 65211, United States; NextGen Precision Health, University of Missouri, Columbia, MO 65211, United States; Laboratory for BioMolecular Structure (LBMS), Brookhaven National Laboratory, Upton, NY 11973, United States; Department of Electrical Engineering and Computer Science, University of Missouri, Columbia, MO 65211, United States; NextGen Precision Health, University of Missouri, Columbia, MO 65211, United States

## Abstract

**Motivation:**

Cryogenic electron microscopy (cryo-EM) is a core experimental technique used to determine the structure of macromolecules such as proteins. However, the effectiveness of cryo-EM is often hindered by the noise and missing density values in cryo-EM density maps caused by experimental conditions such as low contrast and conformational heterogeneity. Although various global and local map-sharpening techniques are widely employed to improve cryo-EM density maps, it is still challenging to efficiently improve their quality for building better protein structures from them.

**Results:**

In this study, we introduce CryoTEN—a 3D UNETR++ style transformer to improve cryo-EM maps effectively. CryoTEN is trained using a diverse set of 1295 cryo-EM maps as inputs and their corresponding simulated maps generated from known protein structures as targets. An independent test set containing 150 maps is used to evaluate CryoTEN, and the results demonstrate that it can robustly enhance the quality of cryo-EM density maps. In addition, automatic *de novo* protein structure modeling shows that protein structures built from the density maps processed by CryoTEN have substantially better quality than those built from the original maps. Compared to the existing state-of-the-art deep learning methods for enhancing cryo-EM density maps, CryoTEN ranks second in improving the quality of density maps, while running >10 times faster and requiring much less GPU memory than them.

**Availability and implementation:**

The source code and data are freely available at https://github.com/jianlin-cheng/cryoten.

## 1 Introduction

In a cryogenic electron microscopy (cryo-EM) experiment, purified proteins in solutions are fast frozen at cryogenic temperature and then imaged by an electron microscope to obtain their structural information. Compared to traditional techniques (i.e. X-ray crystallography and nuclear magnetic resonance), cryo-EM has the unique capability of determining the atomic structures of large protein complexes and assemblies consisting of multiple protein chains, which are difficult or impossible for other techniques to handle. The reconstruction of protein structures from cryo-EM maps involves three main steps: picking protein particles in 2D cryo-EM micrographs (Bepler *et al.* 2020, Dhakal *et al.* 2024, Gyawali *et al.* 2024, Dhakal *et al.* 2025), aligning the 2D protein particle images from different orientations to reconstruct 3D electron density map (Punjani *et al.* 2017, Zhong *et al.* 2021), and using the map for *de novo* atomic structural model building (Terashi and Kihara 2018, Terwilliger *et al.* 2018a, Giri and Cheng 2024). However, one of the main factors affecting the effectiveness of this process is the low contrast and noise present in the 3D electron density map. This problem is partially addressed by various post-processing techniques such as global and local map sharpening that modify the density values of the cryo-EM maps (Rosenthal and Henderson 2003, Scheres 2012, Terwilliger *et al.* 2018b).

Global map sharpening generally involves applying a single B-factor correction to the entire map, aiming to restore contrast and improve interpretability (i.e. the amount of structural information in the map determines the quality of protein structures built from the map). Similar global map-sharpening techniques are implemented in phenix.auto_sharpen (Terwilliger *et al.* 2018b), RELION (Rosenthal and Henderson 2003, Scheres 2012) post-processing and CryoSPARC (Punjani *et al.* 2017) sharpening tools. However, assuming a uniform B-factor across the entire map may fail to adapt to the local variations in the map. This could lead to some regions being under-sharpened and other regions being over-sharpened, and cause poor interpretability. Local map-sharpening addresses this shortcoming by taking the local variations in cryo-EM map into consideration and adjusting the local regions in the density map accordingly. LocalDeblur (Ramírez-Aportela *et al.* 2020) uses a Wiener filter-based local deblurring on the cryo-EM density map with a strength proportional to the pre-computed local resolution in each region of the map. LocSpiral (Kaur *et al.* 2021) employs the concept of spiral phase transformation to factorize the volume and enhance high-resolution features locally while preventing map distortions. LocScale (Jakobi *et al.* 2017) uses a sliding window approach to locally scale up the amplitudes of the density map to match with a pre-computed atomic reference structure. Although these local map-sharpening techniques have achieved some success, they still have drawbacks. LocScale requires the prior availability of atomic models, a condition that cannot be met most time. LocSpiral and LocalDeblur require a mask to differentiate the noise and macromolecules in the cryo-EM map, which is often not readily available.

To overcome these limitations, deep learning based approaches have been proposed for fully automated cryo-EM map sharpening. DeepEMhancer (Sanchez-Garcia *et al.* 2021) aims to mimic the local map-sharpening effect of LocScale by training their U-Net (Ronneberger *et al.* 2015) based deep neural network using LocScale generated maps. Since the target LocScale generated maps were created from experimental maps, lower quality experimental maps can limit DeepEMhancer’s performance. Therefore, although DeepEMhancer performs well on some experimental cryo-EM maps, it faces difficulty in robustly improving a wide range of cryo-EM maps. In contrast, EMReady (He *et al.* 2023) uses a Swin-Conv-Unet (Zhang *et al.* 2023) based deep neural network trained on simulated maps that were generated from known protein structures using a reference Gaussian function. EMReady trained on the simulated maps can robustly improve the quality and interpretability of a wide range of experimental cryo-EM maps. EM-GAN (Maddhuri Venkata Subramaniya *et al.* 2023) is another deep learning based map-sharpening method that uses simulated maps generated from known protein structures to train its 3D generative adversarial network (GAN). The GAN is trained to modify low resolution input maps into outputs that are indistinguishable from actual high-resolution simulated maps. However, EM-GAN is very slow when compared to other methods (Supplementary Table S3).

Following EMReady and EM-GAN’s approach of using simulated maps generated from known protein structures as labels to train deep learning methods, we developed CryoTEN—a 3D UNETR++ (Shaker *et al.* 2024) style transformer equipped with efficient paired attention (EPA) for enhancing cryo-EM density maps. CryoTEN robustly improves the interpretability of experimental cryo-EM maps. The EPA attention helps in effectively learning both spatial and channel-wise discriminative features. U-Net style skip connections aid in retaining the spatial information between the downsampling and upsampling layers and thus enhance the cryo-EM map without significant loss of information during the encoding and decoding process. CryoTEN was evaluated on a diverse set of experimental cryo-EM primary and half maps using various map-model validation metrics. The results show that the quality of the maps processed by CryoTEN is substantially better than the original experimental cryo-EM maps and can be used to build better structural models than the original ones. In addition to effectively enhancing cryo-EM maps, CryoTEN can also run efficiently by consuming less GPU resources while being significantly faster when compared to existing deep neural network based map-sharpening methods.

## 2 Materials and methods

### 2.1 Data collection

The advanced search tool of the RCSB Protein Data Bank (PDB) was used to filter PDB structures built from deposited single-particle cryo-EM maps that have a resolution between 2 and 7 Å. For PDB structures with multiple associated cryo-EM maps, only one cryo-EM map was chosen. Similarly, for cryo-EM maps with multiple associated PDB structures, only one structure was chosen. PDB structures with non-orthogonal map axes were removed. Protein FASTA sequences and deposited cryo-EM primary maps were fetched for the filtered PDB structures from the PDB and Electron Microscopy Data Bank (EMDB), respectively. We used phenix.map_model_cc (Afonine *et al.* 2018) to compute cross correlation (CC) scores between PDB structures and associated deposited cryo-EM maps. To ensure the quality of the data, only maps that have CC_mask value >0.7 and CC_box value >0.6 were selected. Finally, to remove redundant cryo-EM maps that have similar sequences/structures, we used MMseqs2 (Steinegger and Söding 2017) to cluster their respective PDB structures that have a sequence identity >30%, and only one structure was selected per cluster. The final non-redundant data collection consists of 1521 PDB structure and map pairs. Among the selected 1521 maps, 827 maps contain ligands, 83 maps contain DNA, and 122 maps contain RNA macromolecules in addition to the protein macromolecules. This alleviates potential deleterious effect that could affect experimental cryo-EM maps containing non-protein macromolecule regions. Out of 1521 maps, we randomly selected 1295 maps as the training set, 76 maps as the validation set, and 150 maps as the test set.

### 2.2 Data processing

To train CryoTEN, the deposited experimental cryo-EM primary maps were used as input, and high-quality simulated maps generated from the corresponding PDB structures were used as targets (labels). These target simulated maps (label) were computed from PDB structures using a reference Gaussian function (DiMaio *et al.* 2009) as follows:


(1) $$\rho_{c}(y)=\sum_{\text{atoms}\;i}C\cdot A_{i}\cdot \;\text{exp}\;\left(-k\cdot {\left\|x_{i}-y\right\|}^{2}\right)$$

The density value $\rho_{c}(y)$ for a grid point with coordinate **y** in the simulated map is computed according to its distance with every atom in the PDB structure, where *x_i_* is the coordinates of the *i*th atom in the PDB structure and *A_i_* is the atomic number of the *i*th atom; $k=(\pi /{\left(0.9R_{0}\right)}^{2}$, where *R*_0_ is the unmasked Fourier shell correlation resolution at 0.143 threshold (FSC@0.143) of the deposited cryo-EM primary map computed using phenix.mtriage (Afonine *et al.* 2018) tool; and $C={\left(k/\pi \right)}^{3/2}$. The density value $\rho_{c}$ is computed for all grid points to construct the simulated map. All atoms (including hydrogen and nonstandard residue atoms) present in the PDB structure are modeled as part of the simulated map to alleviate potential deleterious effect on non-protein macromolecule regions.

**Figure 1.**
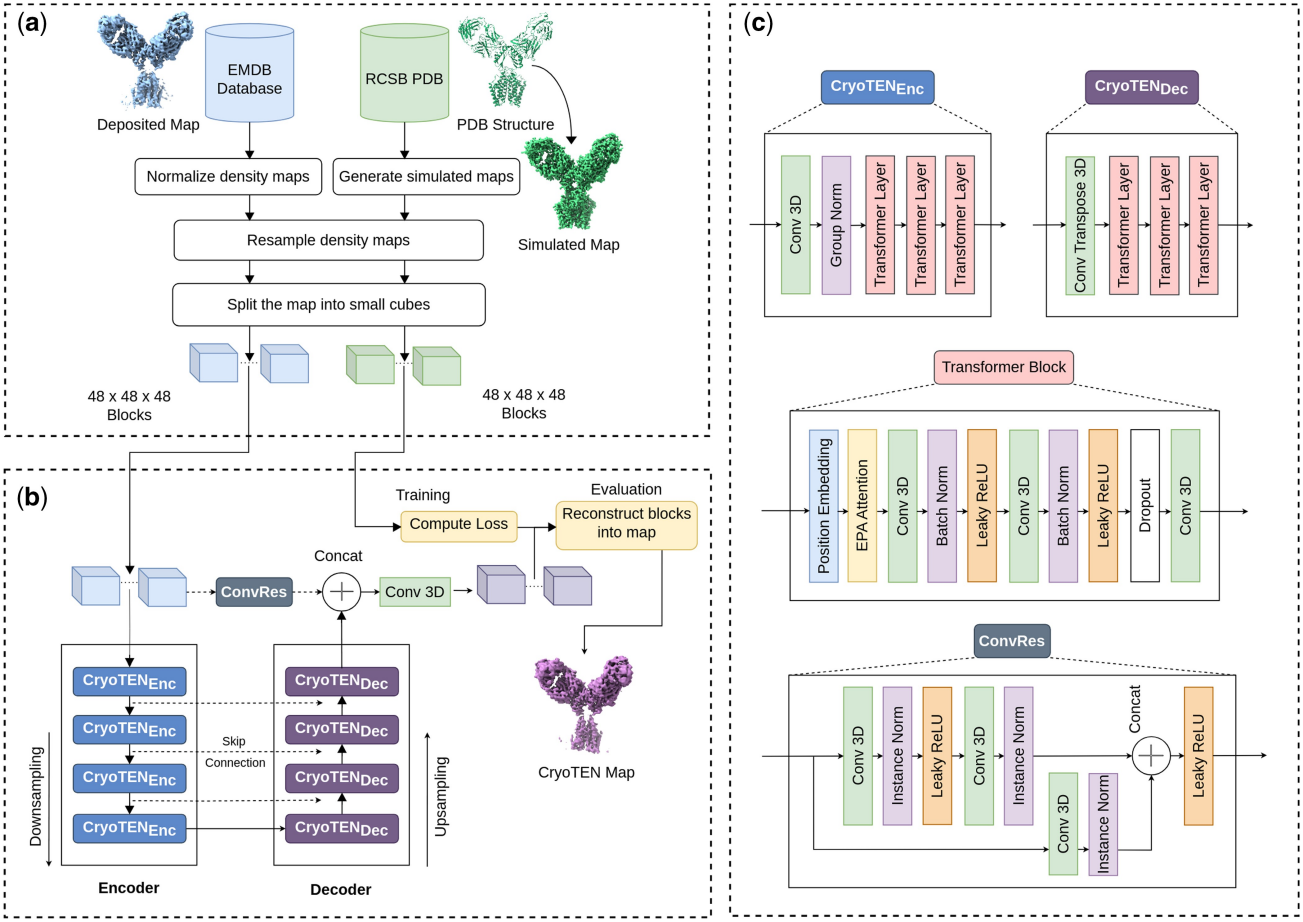
Overview of data processing and CryoTEN model architecture. (a) Data collection and preprocessing. (b) The CryoTEN model architecture along with the training and evaluation pipeline. (c) The structure of the CryoTEN model’s encoder, decoder, and residual convolution (ConvRes) block.

Both the deposited cryo-EM primary maps and simulated density maps were resampled to a common, standardized grid size of 1 Å. Density values of deposited cryo-EM maps were normalized to a range of 0–1 by the 99.999th percentile density value. Since cryo-EM maps are different in size and often large, we split all the maps into smaller blocks (cubes) so that each of them does not require much GPU memory to hold. Specifically, in the training set, deposited cryo-EM primary maps and simulated maps were initially split into overlapping blocks of size 64 × 64 × 64 with a stride length of 50, and were randomly cropped to blocks of size 48 × 48 × 48 on the fly during training to reduce overfitting. Only the blocks that contain protein structures were selected for training, while empty blocks were ignored. In the validation and test set, the maps were directly split into overlapping blocks of fixed size 48 × 48 × 48 with a stride length of 38. An overview of the data preparation process is illustrated in Fig. 1a.

### 2.3 Neural network architecture

CryoTEN is a UNETR++ (Shaker *et al.* 2024) style transformer-based deep neural network. It consists of four pairs of transformer-based encoder (*CryoTEN_Enc_*) and decoder (*CryoTEN_Dec_*) layers with U-Net like skip connections between the encoders and decoders as shown in Fig. 1b. An encoder consists of a down-sampling convolution, group normalization, and three transformer layers. A decoder consists of an upsampling convolution transpose and three transformer layers. The input cryo-EM density map block data are passed through the four encoder layers and then through the four decoder layers. The skip connection between the encoder and decoder layers reduces the loss of spatial information during the encoding and decoding process. During back-propagation, skip connections also provide an alternate path for the gradients to flow which helps in reducing the vanishing or exploding gradient problem in larger neural networks. This makes the training more stable and efficient.

The input cryo-EM density map block data are also passed through an intermediate residual convolution layer called ConvRes (see its structure in Fig. 1c). The output of the ConvRes layer is concatenated with the output of the last decoder layer. The concatenated output is finally passed through a convolutional layer that upsamples the output to the original shape of the input cryo-EM density map block. In the ConvRes layer, the input is passed through two pairs of convolution and normalization layers. This output is then concatenated with a residual connection to the input through a third pair of convolutional and normalization layer. The concatenated output is finally passed through a Leaky ReLU activation layer. Similar to the skip connections, the residual connection within ConvRes layer reduces the loss of information by retaining information from earlier layers to the latter layers.

Transformer layers are equipped with positional embedding, convolution layer, Leaky ReLUs (rectified linear unit), and efficient paired attention (EPA) (Shaker *et al.* 2024) along with batch normalization and dropouts for regularization as shown in Fig. 1c. The dropout layer helps in preventing overfitting and improves the model’s ability to generalize on unseen data. The EPA attention introduced in UNETR++ (Shaker *et al.* 2024) is a novel attention mechanism designed to efficiently learn spatial and channel-wise features. The key idea of EPA attention is to share the weights of the query and key mapping functions between the spatial and channel attention features. This not only reduces the number of parameters but also allows the two branches to complement each other, leading to better feature representation. EPA also achieves better efficiency in spatial attention computation by projecting the key and value mappings to lower dimensional feature vectors before computing the dot product. This enables EPA to perform better while being faster and consuming significantly less GPU memory (Shaker *et al.* 2024). Thus, the UNETR++ based architecture with EPA attention enables CryoTEN to be faster and more efficient than other deep learning based map-sharpening methods.

### 2.4 Experimental setup

CryoTEN was trained for 827 epochs on 4× NVIDIA-A40 GPU. Each NVIDIA-A40 has a GPU memory of 48 GB. One of the key factors in selecting the batch size and the number of encoder and decoder layers of CryoTEN was the available GPU memory. As cryo-EM density maps have different sizes and are often huge, we split them into smaller blocks to fit them into GPU memory. A batch size of 64 blocks and four encoder and decoder layers are optimally chosen to fit the available GPU memory.

During training, as shown in Fig. 1, the blocks of the experimental cryo-EM primary maps were used as input to CryoTEN. The output generated by CryoTEN was compared against the corresponding simulated density blocks (labels) to compute the loss. The loss is calculated using the masked mean squared error (MSE) loss, where voxels being zero in both output blocks and simulated density blocks are masked and the loss for the remaining voxels after masking is calculated using the following equation:


(2) $$\text{loss}=\frac{\sum_{i=1}^{N}{\left(x_{i}-y_{i}\right)}^{2}}{N}$$

where *N* is the total number of voxels after masking, *x_i_* is the value of the *i*th voxel in the output map block and *y_i_* is the value of the *i*th voxel in the simulated density block. The Adam optimizer with an initial learning rate of 0.0005 was used to train the model to minimize the loss.

Several regularization techniques are implemented at various stages of CryoTEN to reduce overfitting and improve generalization. During the data processing stage, a non-redundant set of maps is selected for both training and evaluation by ensuring the maps do not have a sequence identity of >30%. The diverse nature of the dataset and the large number of maps used in training (1295 maps) and evaluation (150 maps) ensures the ability of CryoTEN to generalize well. In the neural network architecture as shown in Fig. 1, a dropout layer is used in the transformer layers present in both the encoder and decoder layers. During training, the dropout layer randomly zeroes some elements in the input tensor with a probability of 0.1. This is an effective regularization technique that prevents co-adaptation of neurons and improves the generalization capability of deep neural networks (Hinton *et al.* 2012). To further reduce over-fitting during training, we augmented the training data by randomly cropping 48 × 48 × 48 density map blocks from 64 × 64 × 64 density map blocks. We also applied random 90° rotation and random axis flipping to the training data to further augment it. The data augmentation techniques ensure that the same input data is not seen often during each epoch of the training and thus enable the model to be trained for longer epochs without overfitting. The model was validated on the validation set for four times per epoch, and the learning rate was halved till 0.00001 if the validation loss did not improve in four consecutive times. This helps the model to converge faster (with an initially high learning rate) and aids in finding better minima by dynamically reducing the learning rate. These regularization techniques performed at various stages of CryoTEN development enable the model to be trained longer while avoiding overfitting and generalizing well. During the training process, at different epochs, we evaluated CryoTEN using the map-model validation metrics on a subsample of the validation set maps and the model was trained till a notable improvement in the metrics was observed.

## 3 Results

After training and validation, we blindly evaluated CryoTEN on the independent test set. During the evaluation, each input cryo-EM map was sliced into 48 × 48 × 48 blocks, which were fed as input to CryoTEN in batches. The output blocks generated by CryoTEN were then assembled to reconstruct the enhanced full density map that has the same size as the input density map. The quality of the enhanced maps was then assessed.

### 3.1 Map-model validation metrics

Map-model validation metrics (Afonine *et al.* 2018, Lawson *et al.* 2021) are essential tools that provide quantitative measures of how well the model agrees with the experimental map and help identify potential errors or areas of uncertainty in the model or map. In our evaluations, we first compute various map-model validation metrics between the experimental cryo-EM maps (before enhancing with CryoTEN) and their corresponding known protein structure and then compare it to the map-model validation metrics computed between CryoTEN enhanced cyro-EM maps and the respective known protein structures. This provides a comprehensive analysis of how well the experimental cryo-EM maps and CryoTEN enhanced maps relate to the atomic model of the known protein structures. The details of each map-model validation metric used in our evaluation process are discussed below.

The map-model Fourier shell correlation (FSC) is computed using the phenix.mtriage (Afonine *et al.* 2018) tool and the unmasked FSC resolution at both 0.143 (FSC@0.143) and 0.5 (FSC@0.5) thresholds are reported in our evaluations. The phenix.mtriage tool creates a simulated map from the atomic model called the model-calculated map. The map-model FSC measures the correlation between the experimental cryo-EM map and the model-calculated map at different spatial frequencies in the Fourier space. The map-model FSC resolution is different from the widely reported gold-standard FSC resolution. The gold-standard FSC resolution is calculated between two independent half maps generated from two halves of the experimental dataset and measures the reproducibility and consistency of the map features derived from the independent reconstructions. In contradiction, the map-model FSC resolution computed between the experimental map and the atomic model denotes how well the atomic model correlates with the experimental cryo-EM map. Thus, the map-model FSC resolution values should not be one-to-one compared with the reported gold-standard FSC resolution values.

We also compute three map-model CC scores: CC_box, CC_mask, and CC_peaks using the phenix.map_model_cc (Afonine *et al.* 2018) tool. The phenix.map_model_cc tool creates a molecular mask around the macromolecule (the known protein structure). It also builds a simulated map from the known protein structure called the model-calculated map. CC_box is the cross-correlation computed between the entire experimental map and the model-calculated map. A high CC_box value indicates that both the regions containing the macromolecule and the regions outside the macromolecule are well correlated with the atomic model, which may also indicate a better signal-to-noise ratio between the experimental map and model. CC_mask is computed between the experimental and model-calculated maps for regions only inside the molecular mask (i.e. the region containing the macromolecular structure). A higher CC_mask score signifies the atoms in the model are well positioned within the experimental map. CC_peaks is computed around the union of regions having the *N* highest density values in the model-calculated and experimental maps, where *N* is the number of grid points present inside the molecular mask. This denotes that high-density regions often containing concentrations of heavy atoms agree well between the model and experimental map. Together, the three cross-correlation scores provide an overlook on the quality of the cryo-EM map both in and around the macromolecular regions.

Finally, to measure the resolvability of atoms in the maps, the *Q*-score (Pintilie *et al.* 2020) metric is computed using UCSF Chimera (Pettersen *et al.* 2004) mapq plugin. *Q*-score is calculated by comparing the density values around each atom in the experimental map with the ideal/expected density values computed based on the map’s overall resolution. A high *Q*-score (close to 1) indicates that the density around the atom is well-defined and matches the expected density for the map’s resolution and also suggests a high confidence in the atom’s position.

### 3.2 Evaluation on cryo-EM primary maps

CryoTEN is first evaluated on the test set containing 150 cryo-EM deposited primary maps using the various map-model validation metrics discussed above. The average scores of these metrics for the original deposited primary maps and the CryoTEN processed maps are reported in Table 1.

**Table 1.** The comparison of the average map-model validation metrics scores of the 150 deposited cryo-EM primary maps in the test dataset and the corresponding CryoTEN processed maps.

Metrics	Deposited	CryoTEN
FSC@0.143 (Å)	3.55	2.48
FSC@0.5 (Å)	6.38	3.79
CC_Box	0.7231	0.8512
CC_Mask	0.7874	0.7685
CC_Peaks	0.6439	0.7480
*Q*-score	0.5378	0.5425

The scores are computed by comparing the maps with their respective atomic structures retrieved from the PDB.

The average map-model FSC@0.143 resolution of the CryoTEN processed cryo-EM primary maps is 2.48 Å, 30.14% better than 3.55 Å of the deposited cryo-EM primary maps. Similarly, the average map-model FSC@0.5 resolution of the CryoTEN processed maps is 3.79 Å, 40.60% higher than 6.38 Å for the deposited cryo-EM primary maps. Out of 150 maps, 99.33% of the CryoTEN processed maps exhibit an improvement in terms of the map-model FSC@0.143 resolution, and 95.33% of them show an improvement in terms of the map-model FSC@0.5 resolution. The consistent performance and significantly improved map-model FSC resolutions denote that the CryoTEN enhanced maps have more higher resolution features than the deposited cryo-EM primary maps.

In terms of the CC scores, the CryoTEN processed maps have substantially higher average CC_box and CC_peaks scores than the deposited primary maps, while their average CC_mask score is slightly lower. CryoTEN achieves an average CC_box score of 0.8512, which is 17.72% higher than 0.7231 of the deposited cryo-EM maps and average CC_peaks score of 0.7480, 16.17% higher than 0.6439 of the deposited cryo-EM maps. However, the average CC_Mask score of the CryoTEN processed maps is 0.7685, which is 2.4% lower than 0.7874 of the deposited cryo-EM maps. Although slightly reduced CC_mask may indicate that all the atoms did not fit the CryoTEN enhanced map as well as the deposited cryo-EM primary map, the variation is much less. However, the improved CC_peaks indicates that the high-density regions (that generally encompass heavy atoms) in CryoTEN map are significantly well-defined than the deposited cryo-EM primary. An improved CC_box indicates an overall improvement in the map quality and a reduction in noise outside the macromolecular region. Overall, both regions containing the macromolecule and outside the macromolecule have notably improved in CryoTEN enhanced maps.

Finally, the average *Q*-score of the CryoTEN processed maps is 0.5425, which is marginally better than 0.5378 of the deposited cryo-EM maps indicating a mild improvement in the overall resolvability of atoms in CryoTEN enhanced maps. In conclusion, the significantly improved FSC resolution computed in the Fourier space, cross-correlation scores (CC_box and CC_peaks) computed in real space and mildly improved *Q*-score denotes that CryoTEN can reliably enhance deposited cryo-EM primary maps. Supplementary Figure S1 further compares the distribution of the map-model FSC resolution, *Q*-score, and CC scores between the deposited cryo-EM primary maps and CryoTEN processed maps.

### 3.3 Evaluation on cryo-EM half maps

During the 3D cryo-EM density map reconstruction process, the protein particle images are usually split randomly into two subsets, each of which is used to build one separate 3D EM map called a half map in order to assess the reliability of the reconstruction according to the consistency between the two half maps. The unprocessed cryo-EM half maps tend to have lower resolution and higher noise than the primary maps which are usually post-processed using map-sharpening techniques. Therefore, in addition to evaluating CryoTEN on experimental primary maps that have been processed by the post-processing techniques, here we analyse how well CryoTEN can handle raw cryo-EM half maps that have not been processed by the cryo-EM post-processing techniques at all. Out of the 150 maps in the test set, 70 maps have corresponding unprocessed cryo-EM half map pairs available in EMDB. For each of them, one half map is chosen for the evaluation, resulting in 70 raw half maps for assessing CryoTEN. The results are reported in Supplementary Table S1.

Similar to the results on the primary density map evaluation in the previous section, the CryoTEN processed half maps have much higher average FSC@0.143 resolution, FSC@0.5 resolution, CC_Box score, and CC_Peaks score and moderately higher *Q*-score than the deposited half maps, while their average CC_Mask score is slightly lower. The results show that, although CryoTEN is only trained on the deposited cryo-EM primary maps, it can also enhance unprocessed cryo-EM half maps. Since CryoTEN can enhance both processed and unprocessed cryo-EM maps, it can be used with existing post-processing tools such as RELION (Rosenthal and Henderson 2003, Scheres 2012) and CryoSPARC (Punjani *et al.* 2017) to improve the quality of any kind of density maps. Supplementary Figure S2 further compares the distributions of the FSC resolutions, *Q*-score, and CC scores of the deposited cryo-EM half maps and CryoTEN processed half maps.

Traditional map-sharpening methods such as B-factor based sharpening and Wiener filtering have controlled modification on the density values and preserve the underlying density distribution characteristics. However, deep neural network based methods such as CryoTEN, may potentially modify the density values in ways that may not reflect the original experimental map in certain cases. Therefore, we do not recommend depositing CryoTEN enhanced maps in public databases such as EMDB and using them for calculating map resolution. The CryoTEN improved maps however can aid in *de novo* structure modeling as discussed in the next section.

### 3.4 Improvement of protein structure modeling (map interpretability)

To evaluate the impact of CryoTEN on improving the quality of protein structures built from cryo-EM density maps (map interpretability), we performed chain-wise structural modeling on deposited cryo-EM primary maps in the test dataset and the corresponding CryoTEN processed primary maps using automatic de novo model building tools, phenix.map_to_model (Terwilliger *et al.* 2018a, Liebschner *et al.* 2019) and MAINMAST (Terashi and Kihara 2018). We used the zone tool in UCSF ChimeraX (Pettersen *et al.* 2021) to extract chain-wise map regions from the maps. The phenix.map_to_model tool automatically builds an atomic model by using the extracted chain-wise map regions, the map’s reported FSC resolution and corresponding chain’s FASTA sequence. We used the default parameters for the phenix.map_to_model computations. In our MAINMAST evaluation, we first traced the main-chain path using only the extracted chain-wise map regions, then used the ThreadCA tool provided in MAINMAST software to thread the sequence file on the predicted main-chain path to get the final atomic model. During main-chain path tracing, for each protein chain we ran MAINMAST with 144 different parameter combinations and for each modeled path, we ran ThreadCA to build the atomic model using the default parameters and respective sequence information of the chain. The atomic model achieving the lowest RMSD (root mean square deviation) score when compared to the deposited protein structure is selected as the final model for the given chain. The parameter combination for MAINMAST path tracing was selected as recommended in the original paper (Terashi and Kihara 2018) with an exception for the density threshold parameter. For deposited cryo-EM maps, we used a combination of [author recommended contour level ×0.25, ×0.5, ×0.75, and ×1.00] as the density threshold parameters. For CryoTEN enhanced maps, we computed the mean (*μ*) and standard deviation (*σ*) for each map and used a combination of [μ−0.5σ,μ,μ+0.5σ,μ+σ] as the density threshold parameters. To eliminate human influence, the modeled structures of the chains built from deposited maps and the corresponding CryoTEN processed maps are evaluated without any further model refinement.

The generated atomic model for each chain is evaluated using phenix.chain_comparison tool (Liebschner *et al.* 2019) by comparing it against the respective structure of the chain from the PDB (considered as ground truth). The average residue coverage score and sequence match score over all the chains are reported in Table 2. The residue coverage score (%) is the percentage of C*α* atoms in a modeled structure that are aligned within 3 Å to its corresponding PDB structures. The sequence match score (%) is the percentage of residues in the modeled structure whose amino acid types exactly match those in the corresponding PDB structure.

**Table 2.** The quality (residue coverage and sequence match scores) of structural models of chains built from the deposited cryo-EM density maps and the CryoTEN processed maps using phenix.map_to_model and MAINMAST tool.

Maps	phenix.map_to_model		MAINMAST	
	Residue coverage (%)	Sequence match (%)	Residue coverage (%)	Sequence match (%)
Deposited	61.87	34.37	72.31	10.75
CryoTEN	70.74	37.38	80.0	13.8

phenix.map_to_model was computed on 655 chains from 114 maps and MAINMAST was computed on 65 chains from 34 maps.

The quality (residue coverage and sequence match scores) of structural models built using phenix.map_to_model and MAINMAST from CryoTEN processed maps shows consistent improvements over models built from deposited maps (Table 2). For phenix.map_to_model, residue coverage increases from 61.87% (deposited maps) to 70.74% (CryoTEN maps), an 8.87% point improvement, while sequence match improves from 34.37% to 37.38%. Similarly, for MAINMAST, residue coverage rises from 72.31% to 80%, and sequence match improves from 10.75% to 13.8%. These results highlight CryoTEN’s ability to enhance both the placement of C*α* atoms and the accuracy of amino acid identification across different modeling tools.

To evaluate CryoTEN’s performance across different resolution ranges, we analysed models (phenix.map_to_model) built from low (4.5 to 7 Å), medium (3 to 4.5 Å), and high (2 to 3 Å) resolution maps and the results are provided in Supplementary Table S2. We can observe that models built from CryoTEN enhanced maps achieve a better residue coverage and sequence match than models built from deposited cryo-EM primary maps across all resolution ranges with the exception of slightly lesser sequence match for models built using low resolution map regions. To visualize the improvements when performing structure modeling, in Supplementary Fig. S3, we provided five examples of chain-wise structure models built using phenix.map_to_model tool on deposited cryo-EM map regions before and after enhancing with CryoTEN. We can clearly observe that CryoTEN enhanced maps can aid in better *de novo* structure modeling. These results demonstrate that CryoTEN can robustly improve the quality of cryo-EM density maps for generating better structural models. However, it should be noted that here we only perform automatic *de novo* structure modeling to analyse the effects of CryoTEN enhancement of cryo-EM density maps. Further refinement of the structural models by human experts still plays a crucial role in building high-quality atomic models as fully automated density map-based structure modeling methods that match the quality of expert-built models are still not available despite the significant progress (Giri and Cheng 2024) in the area.

### 3.5 Visual validation of CryoTEN enhanced maps

**Figure 2.**
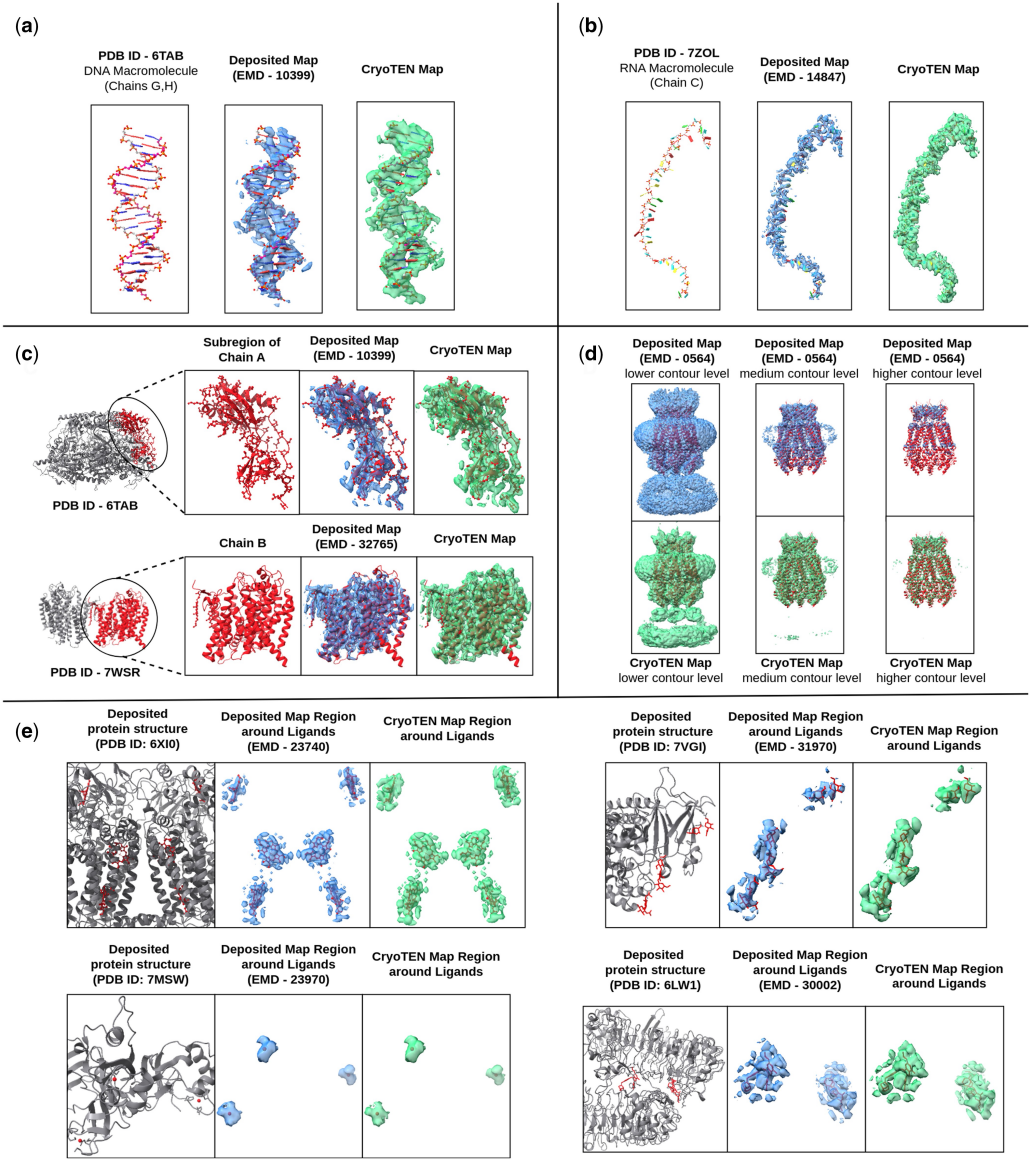
(a) Deposited cryo-EM map (EMD-10399) and CryoTEN enhanced counterpart superimposed with the known DNA macromolecular structure (PDB ID: 6TAB, Chains G and H). (b) Deposited cryo-EM map (EMD-14847) and CryoTEN enhanced counterpart superimposed with the known RNA macromolecular structure (PDB ID: 7ZOL, Chain C). (c) Two examples of deposited (EMD-10399, EMD-32765) and CryoTEN map regions consisting of *α* helices, *β* sheets and loops present in their respective known protein structure (PDB ID: 6TAB, PDB ID: 7WSR). (d) The deposited cryo-EM map (EMD-0564) of the DCPIB-inhibited volume-regulated anion channel LRRC8A in lipid nanodiscs and CryoTEN enhanced counterpart at three different contour levels. (e) Four examples of deposited (EMD-23740, EMD-31970, EMD-23970, EMD-30002) and respective CryoTEN map regions around ligands present in their respective known protein structure (PDB ID: 6XI0, PDB ID: 7VGI, PDB ID: 7MSW, PDB ID: 6LW1) .

In Fig. 2, we present several examples of map regions of deposited experimental cryo-EM maps and CryoTEN enhanced counterparts. The deposited cryo-EM and CryoTEN maps are compared to each other at an equivalent contour level after density normalization.

In the first example (Fig. 2a), we can visualize the deposited and CryoTEN map regions of the DNA macromolecule present within the FtsK protein (PDB ID 6TAB, Chains G and H). In the second example (Fig. 2b), we can visualize the deposited and CryoTEN map regions of the RNA macromolecule present within the protein (PDB ID: 7ZOL, Chain C). In the third example (Fig. 2c), the deposited and CryoTEN map regions consisting of *α* helices, *β* sheets, and loops in a subregion of the FtsK protein (PDB ID 6TAB, Chain A) and barley Yellow stripe 1 transporter protein (PDB ID: 7WSR, Chain B) are shown. In the fourth example (Fig. 2d), we can visualize the deposited cryo-EM map (EMD-0564) of the DCPIB-inhibited volume-regulated anion channel LRRC8A in lipid nanodiscs and CryoTEN enhanced counterpart (green) at three different contour levels. Finally in Fig. 2e, we observe four examples of deposited (EMD-23740, EMD-31970, EMD-23970, EMD-30002) and CryoTEN map regions around ligands present in their respective known protein structure (PDB ID: 6XI0, PDB ID: 7VGI, PDB ID: 7MSW, PDB ID: 6LW1). From the above examples, we can clearly observe that CryoTEN can robustly handle cryo-EM maps including those that contain nanodiscs, DNA, RNA, and ligand molecules.

In Supplementary Fig. S4, we show an example of CryoTEN substantially improving the deposited cryo-EM map (EMD-22338) of Epstein-Barr virus-encoded G protein-coupled receptor BILF1 at various contour levels by removing noise and enhancing structural details. The deposited maps and the CryoTEN enhanced maps are overlaid with their corresponding protein structure (PDB ID: 7JHJ) to visualize their quality. The regions where CryoTEN makes substantial improvement are circled. For instance, at a higher contour level, the circled high-density regions in the CryoTEN enhanced map match the protein structure better than the deposited map in which some structural details are missing, while at a lower contour level, CryoTEN effectively reduces the background noise in the circled region. A similar example (EMD-22937 of H1 A/Michigan/45/2015 ectodomain) is illustrated in Supplementary Fig. S5. Specifically, in the case of EMD-22338, the structural model of Chain R built from the CryoTEN enhanced map has a residue coverage of 78.3%, which is higher than 73.1% from the deposited cryo-EM map. Similarly, the residue coverage of Chains A and D for the former is 90.4% and 86.3%, respectively, substantially higher than 61.9%, and 48.5% for the latter. Further in Supplementary Fig. S3, we can visualize five more examples of chain-wise structure modeling performed on deposited cryo-EM maps before and after enhancing with CryoTEN using phenix.map_to_model tool. These examples clearly illustrate how CryoTEN enhanced density maps can aid model building tools such as phenix.map_to_model to build better protein structures.

### 3.6 Comparison with other deep learning methods

We compare CryoTEN with three existing deep learning methods for enhancing cryo-EM density maps, DeepEMhancer (Sanchez-Garcia *et al.* 2021), EMReady (He *et al.* 2023), and EM-GAN (Maddhuri Venkata Subramaniya *et al.* 2023) on the test dataset in terms of quality of the density maps, empirical time complexity, and memory consumption (Supplementary Table S3). While CryoTEN and EMReady processed all 150 maps in our test set, DeepEMhancer and EM-GAN were able to process only 131 and 146 maps without crashing, respectively. Therefore, to make a fair comparison, we compare average map-model validation metrics on 130 maps that were successfully processed by all the methods. We also assess their running time and memory requirement on a subset of 20 test maps.

The results show that EMReady improves the quality of the deposited density maps in terms of all the metrics, CryoTEN in terms of all the metrics except CC_mask, and DeepEMhancer in terms of resolution (FSC@0.143 and FSC@0.5) but not cross-correlation metrics (CC_box, CC_mask, CC_peaks) and *Q*-score. CryoTEN performs substantially better than DeepEMHancer in terms of all the metrics. For instance, the FSC@0.143 and CC_Box of CryoTEN are 2.48 Å and 0.8505, substantially higher than 3.57 Å and 0.7225 of DeepEMhancer. Similarly, CryoTEN performs much better than EM-GAN in terms of all the metrics. However, it should be noted that the EM-GAN results are severely affected by the maps for which EM-GAN has generated poor quality maps. As shown in Supplementary Table S4, removing poor quality EM-GAN maps with negative correlation scores improves the overall scores of all map-validation metrics. However, still CryoTEN performs notably better than EM-GAN in all map-validation metrics. The performance of CryoTEN is relatively close to the best-performing EMReady in terms of all the metrics, and both of them substantially increase FSC@0.143, FSC@0.5, CC_box, and CC_peaks of the maps. EMReady moderately increases the *Q*-score, while CryoTEN only slightly improves it. EMReady has a CC_mask score of 0.7913, slightly higher than 0.7845 of the deposited maps, while CryoTEN has a slightly lower CC_mask score of 0.7674. Among 150 maps in the test dataset, CryoTEN performs better than EMReady in 51 maps in terms of CC_box scores, 31 maps in terms of CC_mask scores, 34 maps in terms of CC_peaks scores, and 26 maps in terms of all three cross-correlation scores. These improvements are limited to certain cases and overall EMReady performs better than CryoTEN in the average map-model validation metrics.

Although CryoTEN performs second best in terms of map-model validation metrics, it runs significantly faster than all the related methods and requires much less GPU memory than EMReady and DeepEMhancer. The execution time and memory consumption of the four deep learning methods (DeepEMhancer, EMReady, EM-GAN, and CryoTEN) were empirically compared on a subset of 20 density maps in the test dataset. On average, CryoTEN takes only 1.66 min to enhance a map with a standard deviation of ±0.42 min, whereas EMReady takes 19.65 min per map with a standard deviation of ±17.95 min, EM-GAN takes 340.41 min per map with a standard deviation of ±300.94 min and DeepEMhancer takes 43.27 min per map with a standard deviation of ±9.41 min. Moreover, on an NVIDIA A10 GPU, with batch size configured as 40 for all methods, CryoTEN consumes only 3.41 GB of GPU memory, compared to 21.88 GB of DeepEMhancer and 8.86 GB of EMReady. Although EM-GAN consumed the lowest GPU memory of 0.8GB, it performed significantly slower than the other methods. The per map runtime and memory consumption for DeepEMhancer, EM-GAN, and EMReady are reported in Supplementary Table S5. This shows the efficiency of the CryoTEN architecture and the EPA attention mechanism used in the transformer layers in the encoder and decoders. Therefore, CryoTEN is suitable for high-throughput enhancement of cryo-EM density maps where the processing speed and computational resources are critical.

## 4 Conclusion

In this study, we introduce CryoTEN, a UNETR++ based transformer model equipped with the EPA attention mechanism to enhance cryo-EM density maps. CryoTEN is trained on a large non-redundant diverse set of 1295 deposited cryo-EM primary maps and extensively evaluated on an independent test set. It can improve the quality of both primary density maps and half density maps in terms of multiple map-model validation metrics. The automatic *de novo* structure modeling experiment shows that the CryoTEN processed maps can be used to build better protein structural models than the original density maps. Moreover, CryoTEN runs more than 10 times faster and requires much less GPU memory than the existing deep learning methods, while still achieving a rather good performance in enhancing the quality of cryo-EM density maps.

Albeit CryoTEN’s reliable performance, it should be noted that, unlike traditional techniques like B-factor map sharpening that modifies the amplitude in Fourier space, deep neural network based methods such as CryoTEN modify the density values directly. This could affect the phase and amplitude of the density map and lead to suboptimal results or misinterpretation of the input data in certain cases. Although we observed that CryoTEN can reliably handle DNA, RNA, nanodiscs, and in many cases even ligands, it may still have deleterious effects on water molecules and ligand regions in certain cases (as shown in Supplementary Fig. S6). Therefore, due to these shortcomings of deep neural network based methods, we would like to clarify that CryoTEN enhanced maps are intended to only aid in *de novo* structure modeling and should not to be used for other purposes such as EMDB deposition and FSC map resolution. We recommend users to carefully validate when modeling structure using a CryoTEN processed map and consider further refining the model using the original experimental map if needed. In the future, with increase in more high quality deposited maps containing other molecule types such ligands and water molecules, deep neural network based approach may evolve to handle them reliably.

## Supplementary Material

btaf092_Supplementary_Data

## Data Availability

The fine-tuned model weights are available at https://zenodo.org/records/14736781/files/cryoten_v2.ckpt. The dataset used for training and evaluating CryoTEN can be downloaded using the scripts provided in our GitHub repository: https://github.com/jianlin-cheng/cryoten.

## References

[btaf092-B1] Afonine PV , Klaholz BP, Moriarty NW et al New tools for the analysis and validation of cryo-EM maps and atomic models. Acta Crystallogr D Struct Biol 2018;74:814–40. 10.1107/s2059798318009324 30198894 PMC6130467

[btaf092-B2] Bepler T , Kelley K, Noble AJ et al Topaz-Denoise: general deep denoising models for cryoEM and cryoET. Nat Commun 2020;11:5208. 10.1038/s41467-020-18952-1 33060581 PMC7567117

[btaf092-B3] Dhakal A , Gyawali R, Wang L et al CryoTransformer: a transformer model for picking protein particles from cryo-EM micrographs. Bioinformatics 2024;40:btae109. 10.1093/bioinformatics/btae109 PMC10937899 38407301

[btaf092-B4] Dhakal A , Gyawali R, Wang L et al Artificial intelligence in cryo-EM protein particle picking: recent advances and remaining challenges. Brief Bioinform 2025;26:bbaf011. 10.1093/bib/bbaf011 PMC11736895 39820248

[btaf092-B5] DiMaio F , Tyka MD, Baker ML et al Refinement of protein structures into low-resolution density maps using Rosetta. J Mol Biol 2009;392:181–90. 10.1016/j.jmb.2009.07.008 19596339 PMC3899897

[btaf092-B6] Giri N , Cheng J. De novo atomic protein structure modeling for cryoEM density maps using 3D transformer and hmm. Nat Commun 2024;15:5511. 10.1038/s41467-024-49647-6 38951555 PMC11217428

[btaf092-B7] Gyawali R , Dhakal A, Wang L et al CryoSegNet: accurate cryo-EM protein particle picking by integrating the foundational AI image segmentation model and attention-gated U-Net. Brief Bioinform 2024;25;bbae282. 10.1093/bib/bbae282 38860738 PMC11165428

[btaf092-B8] He J , Li T, Huang S-Y. Improvement of cryo-EM maps by simultaneous local and non-local deep learning. Nat Commun 2023;14:3217. 10.1038/s41467-023-39031-1 37270635 PMC10239474

[btaf092-B9] Hinton GE , Srivastava N, Krizhevsky A et al Improving neural networks by preventing co-adaptation of feature detectors. 2012. 10.48550/ARXIV1207.0580

[btaf092-B10] Jakobi AJ , Wilmanns M, Sachse C. Model-based local density sharpening of cryo-EM maps. eLife 2017;6:e27131. 10.7554/eLife.27131 29058676 PMC5679758

[btaf092-B11] Kaur S , Gomez-Blanco J, Khalifa AAZ et al Local computational methods to improve the interpretability and analysis of cryo-EM maps. Nat Commun 2021;12:1240. 10.1038/s41467-021-21509-5 33623015 PMC7902670

[btaf092-B12] Lawson CL , Kryshtafovych A, Adams PD Jr, et al Cryo-EM model validation recommendations based on outcomes of the 2019 EMDataResource challenge. Nat Methods 2021;18:156–64. 10.1038/s41592-020-01051-w 33542514 PMC7864804

[btaf092-B13] Liebschner D , Afonine PV, Baker ML et al Macromolecular structure determination using X-rays, neutrons and electrons: recent developments in *Phenix*. Acta Crystallogr D Struct Biol 2019;75:861–77. 10.1107/S2059798319011471 31588918 PMC6778852

[btaf092-B14] Maddhuri Venkata Subramaniya SR , Terashi G, Kihara D. Enhancing cryo-EM maps with 3D deep generative networks for assisting protein structure modeling. Bioinformatics 2023;39:btad494. 10.1093/bioinformatics/btad494 37549063 PMC10444963

[btaf092-B15] Pettersen EF , Goddard TD, Huang CC et al UCSF Chimera – a visualization system for exploratory research and analysis. J Comput Chem 2004;25:1605–12. 10.1002/jcc.20084 15264254

[btaf092-B16] Pettersen EF , Goddard TD, Huang CC et al UCSF ChimeraX: structure visualization for researchers, educators, and developers. Protein Sci 2021;30:70–82. 10.1002/pro.3943 32881101 PMC7737788

[btaf092-B17] Pintilie G , Zhang K, Su Z et al Measurement of atom resolvability in cryo-EM maps with *Q*-scores. Nat Methods 2020;17:328–34. 10.1038/s41592-020-0731-1 32042190 PMC7446556

[btaf092-B18] Punjani A , Rubinstein JL, Fleet DJ et al cryoSPARC: algorithms for rapid unsupervised cryo-EM structure determination. Nat Methods 2017;14:290–6. 10.1038/nmeth.4169 28165473

[btaf092-B19] Ramírez-Aportela E , Vilas JL, Glukhova A et al Automatic local resolution-based sharpening of cryo-EM maps. Bioinformatics 2020;36:765–72. 10.1093/bioinformatics/btz671 31504163 PMC9883678

[btaf092-B20] Ronneberger O , Fischer P, Brox T. U-net: convolutional networks for biomedical image segmentation. In: Navab N, Hornegger J, Wells WM, Frangi AF (eds.), Medical Image Computing and Computer-Assisted Intervention—MICCAI 2015. Cham: Springer International Publishing, 2015, 234–241.

[btaf092-B21] Rosenthal PB , Henderson R. Optimal determination of particle orientation, absolute hand, and contrast loss in single-particle electron cryomicroscopy. J Mol Biol 2003;333:721–45. 10.1016/j.jmb.2003.07.013 14568533

[btaf092-B22] Sanchez-Garcia R , Gomez-Blanco J, Cuervo A et al DeepEMhancer: a deep learning solution for cryo-EM volume post-processing. Commun Biol 2021;4:874. 10.1038/s42003-021-02399-1 34267316 PMC8282847

[btaf092-B23] Scheres SH. A Bayesian view on cryo-EM structure determination. J Mol Biol 2012;415:406–18. 10.1016/j.jmb.2011.11.010 22100448 PMC3314964

[btaf092-B24] Shaker A , Maaz M, Rasheed H et al UNETR++: delving into efficient and accurate 3D medical image segmentation. IEEE Trans Med Imaging 2024;43:3377–90. 10.1109/TMI.2024.3398728 38722726

[btaf092-B25] Steinegger M , Söding J. MMseqs2 enables sensitive protein sequence searching for the analysis of massive data sets. Nat Biotechnol 2017;35:1026–8. 10.1038/nbt.3988 29035372

[btaf092-B26] Terashi G , Kihara D. De novo main-chain modeling for EM maps using MAINMAST. Nat Commun 2018;9:1618. 10.1038/s41467-018-04053-7 29691408 PMC5915429

[btaf092-B27] Terwilliger TC , Adams PD, Afonine PV et al A fully automatic method yielding initial models from high-resolution cryo-electron microscopy maps. Nat Methods 2018a;15:905–8. 10.1038/s41592-018-0173-1 30377346 PMC6214191

[btaf092-B28] Terwilliger TC , Sobolev OV, Afonine PV et al Automated map sharpening by maximization of detail and connectivity. Acta Crystallogr D Struct Biol 2018b;74:545–59. 10.1107/s2059798318004655 29872005 PMC6096490

[btaf092-B29] Zhang K , Li Y, Liang J et al Practical blind image denoising via swin-conv-UNet and data synthesis. Mach Intell Res 2023;20:822–36. 10.1007/s11633-023-1466-0

[btaf092-B30] Zhong ED , Bepler T, Berger B et al CryoDRGN: reconstruction of heterogeneous cryo-EM structures using neural networks. Nat Methods 2021;18:176–85. 10.1038/s41592-020-01049-4 33542510 PMC8183613

