# Supplementary material for: CryoTEN: efficiently enhancing cryo-EM density maps using transformers
Source: Bioinformatics. 2025 Feb 27;41(3):btaf092. doi: 10.1093/bioinformatics/btaf092 (PMC11906401; doi:10.1093/bioinformatics/btaf092)

CryoTEN: Efficiently Enhancing cryo-EM Density Maps using Transformers - Supplementary Information

Joel Selvaraj[1,2], Liguo Wang[3], Jianlin Cheng[1,2,*]

1. Department of Electrical Engineering and Computer Science, University of Missouri, Columbia - 65211, MO, United States.
2. NextGen Precision Health, University of Missouri, Columbia - 65211, MO, United States.
3. Laboratory for BioMolecular Structure (LBMS), Brookhaven National Laboratory, Upton - 11973, NY, United States.

* - corresponding author (chengji@missouri.edu)

Supplementary Figure 1: Plots of map-model validation metrics computed on deposited cryo-EM primary maps and CryoTEN processed maps

The comparison of the map-model validation metrics scores of the 150 deposited cryo-EM primary maps in the test dataset and the corresponding CryoTEN processed maps. Half violin and box plots of **(a)** unmasked FSC@0.143 resolution, **(b)** unmasked FSC@0.5 resolution (7 outliers with FSC@0.5 > 20Å in deposited primary maps are hidden for better visualization), **(c)** average Q-score, and **(d,e,f)** CC_box, CC_mask, CC_peaks scores respectively.

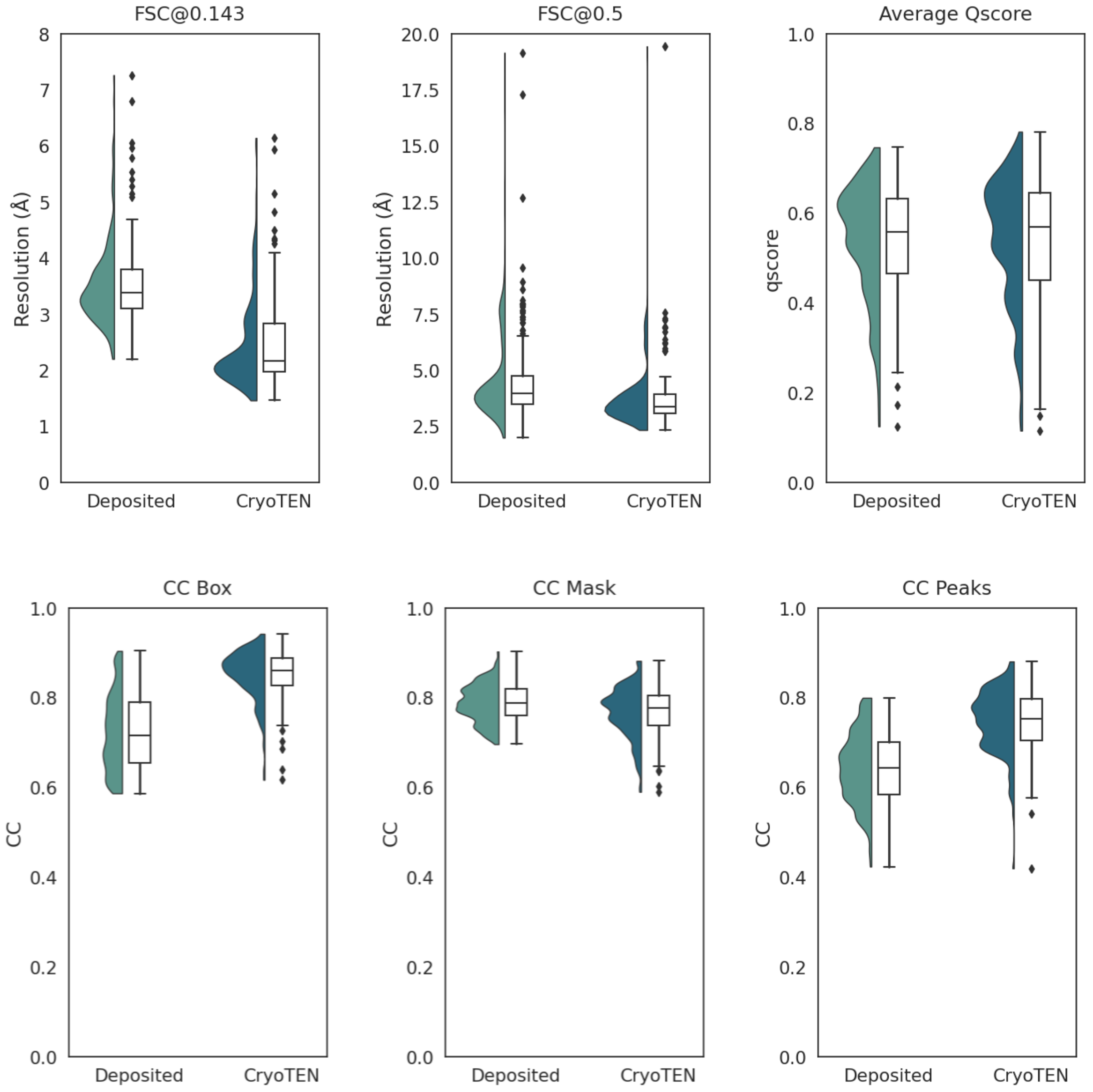

Supplementary Table 1: Comparison of average map-model validation metrics for deposited cryo-EM half maps

Out of the 150 maps in the test set, 70 maps have corresponding unprocessed cryo-EM half map pairs available in EMDB. From each half map pair, half map 1 is chosen for the evaluation, resulting in 70 raw half maps for assessing CryoTEN.

**Metrics**	**Deposited**	**CryoTEN**
FSC@0.143 (Å)	3.75	2.65
FSC@0.5 (Å)	6.2	3.77
CC_Box	0.6796	0.8261
CC_Mask	0.7275	0.7269
CC_Peaks	0.5677	0.7166
Q-score	0.4651	0.4962

Supplementary Figure 2: Plots of map-model validation metrics computed on deposited cryo-EM half maps and CryoTEN processed half maps

Computed various map validation metrics on deposited cryo-EM half maps (one of the half map pairs is used) and CryoTEN processed maps in our test set containing 70 half map pairs. Half violin and box plots of **(a)** unmasked FSC@0.143 resolution, **(b)** unmasked FSC@0.5 resolution (2 outliers with FSC@0.5 > 20Å in deposited half maps are hidden for better visualization), **(c)** average Q-score, **(d,e,f)** CC_box, CC_mask and CC_peaks scores respectively.

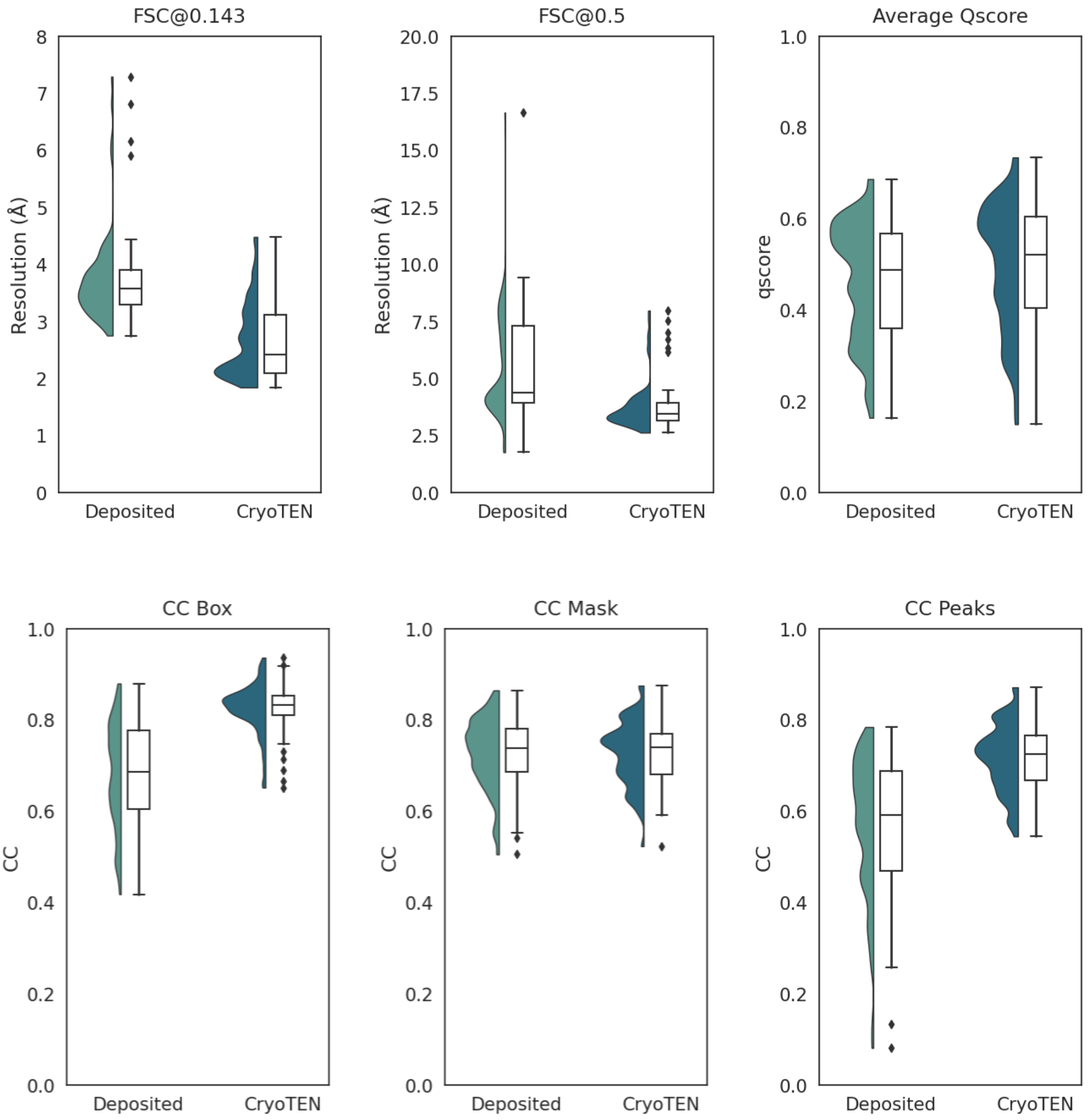

Supplementary Table 2: Robustness in improving map interpretability (structure modeling) for maps of various resolutions

Robustness in improving map interpretability (structure modeling) of maps having various resolutions by CryoTEN. We used the zone tool in UCSF ChimeraX to extract chain-wise map regions from the maps. The extracted chain-wise regions from cryo-EM maps are grouped into three categories based on the map's reported FSC resolution: low, medium, and high resolution, containing 58, 440 and 152 chains respectively. The phenix.map_to_model tool was used to perform automatic de novo structure modeling on the extracted chain-wise map regions using their corresponding FASTA sequence. The phenix.chain_comparison tool was used to compute residue coverage and sequence match scores of the chainwise structural models built from the deposited cryo-EM maps and their CryoTEN enhanced counterparts by comparing them against the known protein structure deposited in PDB. The average scores for each resolution category is reported.

Map Resolution	Method	Residue Coverage(%)	Sequence Match(%)
Low	Deposited	56.18	14.38
(4.5Å to 7Å)	CryoTEN	58.72	11.73
Medium	Deposited	59.06	31.98
(3Å to 4.5Å)	CryoTEN	69.95	36.21
High	Deposited	73.72	49.79
(2Å to 3Å)	CryoTEN	79.4	51.32

Supplementary Figure 3: Visual validation of improvements in map-interpretability

We visualize five different examples of chain-wise structure modeling performed on deposited cryo-EM maps before and after enhancing with CryoTEN using phenix.map_to_model tool. We can observe that CryoTEN enhanced maps lead to better initial structures when performing automatic de-novo structure modeling. The deposited cryo-EM and CryoTEN maps are visualized at an equivalent contour level after performing density normalization.

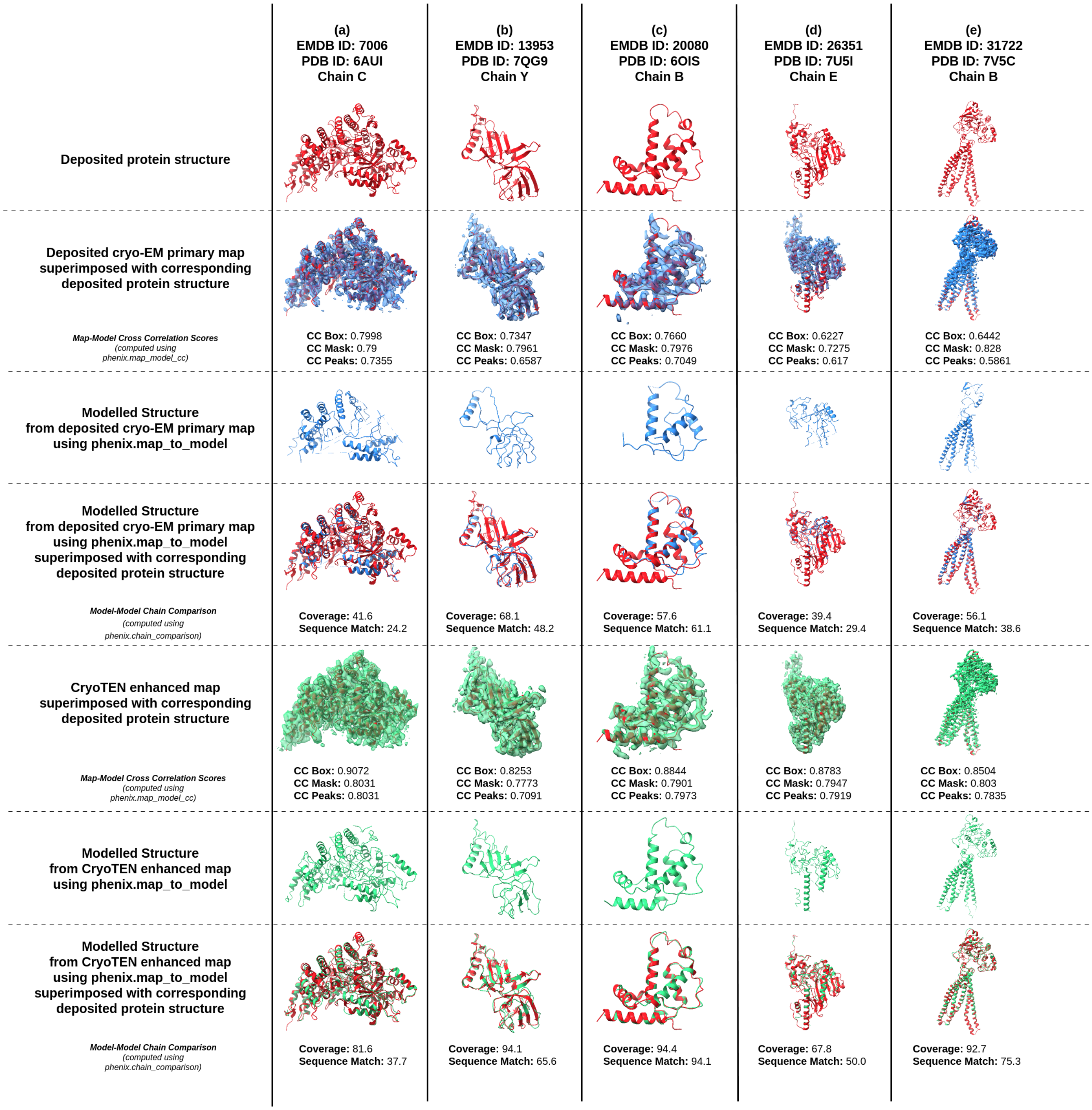

Supplementary Figure 4: First example showing improvement in CryoTEN processed maps

Compares deposited cryo-EM map (EMD-22338)(blue) and its CryoTEN enhanced counterpart (green) in superimposition with the known protein structure (PDB ID: 7JHJ) at three contour levels (lower, recommended, and higher). For direct comparison, the density volume of the two kinds of maps is set to equal at each contour level by adjusting the contour level of the CryoTEN enhanced map. The adjustment of the contour level is needed because the density values in the deposited map and the CryoTEN enhance map are not in the exact same range. The dotted circles in the images of the maps highlight the regions where CryoTEN substantially removes noise and/or adds more structural details.

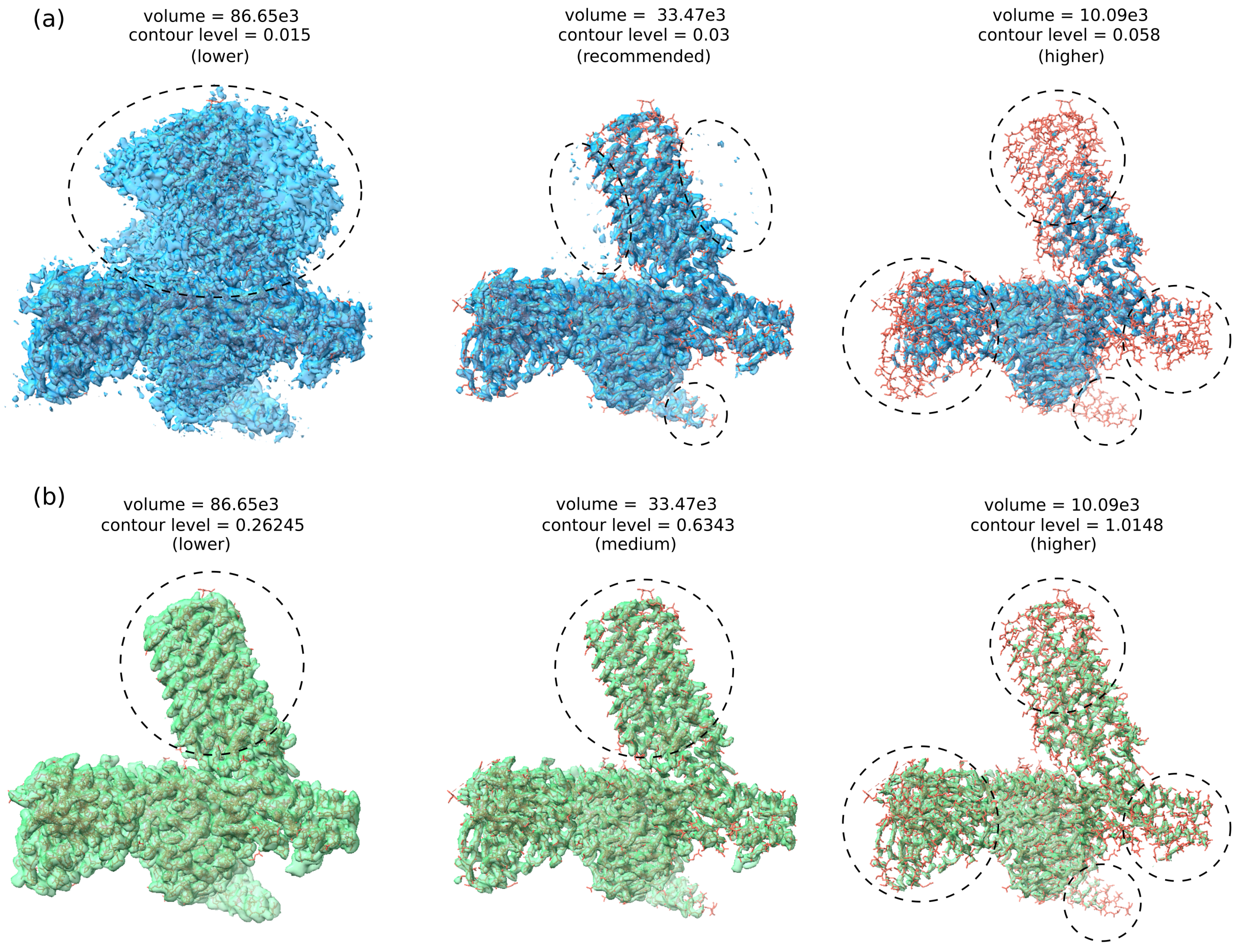

Supplementary Figure 5: Second example showing improvement in CryoTEN processed maps

An example that shows improvement in map quality when using CryoTEN. (a) and (b) compares the deposited density map (blue) and CryoTEN enhanced map (green) of EMD-22937 at various contour levels respectively. Each map overlapped with the corresponding PDB structure (PDB ID: 7KNA) (red). Since the density value distribution varies between maps, we match the density volume between the deposited and CryoTEN processed maps to make a fair comparison. The dotted circles in the images of the maps highlight the regions where CryoTEN substantially removes noise and/or adds more structural detail.

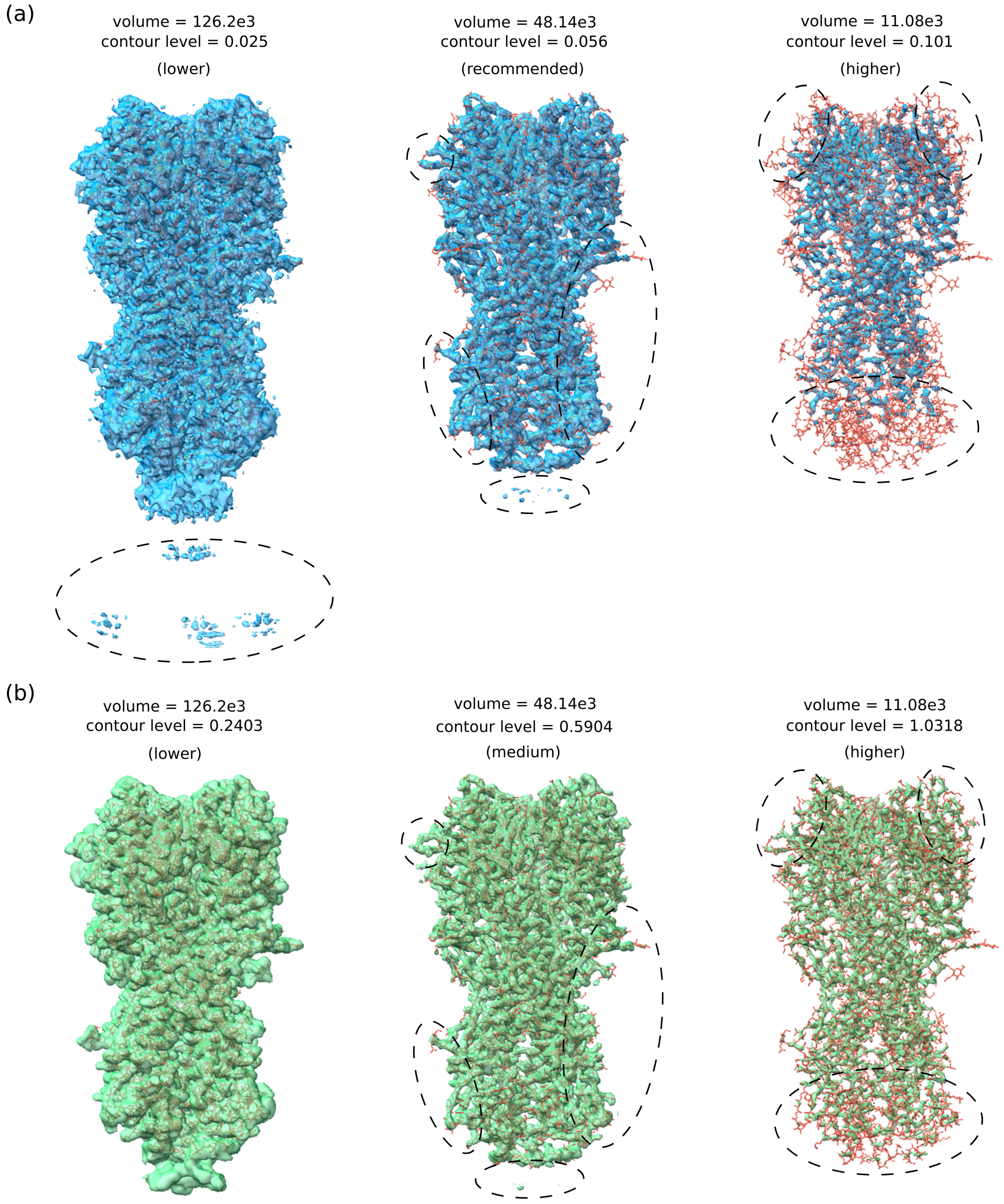

Supplementary Table 3: Comparison with other related methods

Comparison of map-model validation metrics scores of 130 deposited cryo-EM primary maps in the test dataset, DeepEMhancer enhanced maps, EMReady enhanced maps, EM-GAN enhanced maps and CryoTEN enhanced maps as well as the processing time and GPU memory consumption of the three deep learning methods. The average processing time and GPU memory consumption for all three methods is benchmarked on the same subset of 20 maps with a batch size of 40.

**Metrics**	**Deposited**	**DeepEMHancer**	**EMReady**	**EM-GAN**	**CryoTEN**
FSC@0.143 (Å)	3.57	3.53	1.95	4.41	2.48
FSC@0.5 (Å)	5.6	4.58	3.17	6.03	3.83
CC_Box	0.7225	0.6307	0.8620	0.5228	0.8505
CC_Mask	0.7845	0.6092	0.7913	0.5184	0.7674
CC_Peaks	0.6385	0.5728	0.7685	0.3004	0.7443
Q-score	0.5315	0.4248	0.5871	0.4059	0.5359
Avg Processing Time (mins)	-	43.27 ± 9.41	19.65 ± 17.95	340.41 ± 300.94	1.66 ± 0.42
GPU Memory Consumption (GB)	-	21.88	8.86	0.8	3.41

Supplementary Table 4: Comparison with other related methods (Poor quality EM-GAN maps filtered)

Poor quality EM-GAN maps with negative correlation scores are filtered from the test dataset leading to only 96 maps processed successfully by all methods. Comparison of map-model validation metrics scores of 96 deposited cryo-EM primary maps in the test dataset, DeepEMhancer enhanced maps, EMReady enhanced maps, EM-GAN enhanced maps and CryoTEN enhanced maps.

Metrics	Deposited	DeepEMHancer	EMReady	EM-GAN	CryoTEN
FSC@0.143 (Å)	3.57	3.76	1.90	3.11	2.45
FSC@0.5 (Å)	5.96	4.65	3.12	7.58	3.83
CC_Box	0.7194	0.6458	0.8633	0.6663	0.8496
CC_Mask	0.7861	0.6228	0.7921	0.6968	0.7664
CC_Peaks	0.6369	0.5905	0.7713	0.5156	0.7450
Q-score	0.5340	0.4306	0.5927	0.5087	0.5382

Supplementary Table 5: Runtime Benchmark of CryoTEN, DeepEMHancer, EM-GAN and EM-Ready on 20 maps

We ran CryoTEN, DeepEMhancer, EM-GAN and EMReady models on 20 maps in the test set with a batch size of 40 on a NVIDIA A10 GPU. The time taken to process each map by these models is tabulated below. From the total time taken to process 20 maps, we can observe that CryoTEN performed significantly faster than DeepEMhancer, EM-GAN and EMReady.

EMDB ID	CryoTEN Time-Taken (mins)	DeepEMhancer Time-Taken (mins)	EMReady Time-Taken (mins)	EM-GAN Time-Taken (mins)
35363	2.010357	54.836444	14.353148	490.537050
26831	1.214203	37.408689	1.209833	12.877245
27630	1.569806	38.069682	32.110613	331.563632
23092	1.492781	48.146252	8.832931	467.257070
13867	1.193822	41.560315	30.045555	162.201882
23970	1.173997	38.321360	20.812409	118.688424
26978	2.143782	37.432961	5.202853	24.436208
22884	1.896650	54.818219	2.000178	29.135679
32765	2.644996	47.408696	61.382500	468.672467
24784	1.632092	42.320093	31.831395	173.752088
32328	1.292059	40.041838	2.611483	26.638658
4032	2.155701	70.056347	22.552169	1092.354414
26806	1.557495	45.533855	6.369265	468.344539
20226	1.344403	35.729695	5.676419	300.809084
33242	1.560156	34.966081	36.235096	430.238960
14847	1.303207	47.767493	7.853368	796.430868
34369	1.601426	35.538966	25.820971	137.013319
10800	2.093365	50.292193	16.810930	757.987027
15531	1.230027	28.490879	2.447058	8.424245
32940	2.177127	36.571903	58.840337	510.914954
**Total (mins)**	33.29	865.31	393.0	6808.28

Supplementary Figure 6: Limitations of CryoTEN

We visualize deposited cryo-EM map of EMD-26741 along with its CryoTEN enhanced and EMReady enhanced counterparts. The maps are superimposed with their known protein structure (PDB ID: 7USW) and the ligands present in the structure are highlighted in red. The deposited cryo-EM, EMReady and CryoTEN maps are visualized at an equivalent contour level after performing density normalization. We can observe that both CryoTEN and EMReady enhanced maps fails to handle the ligand regions well in this particular case.

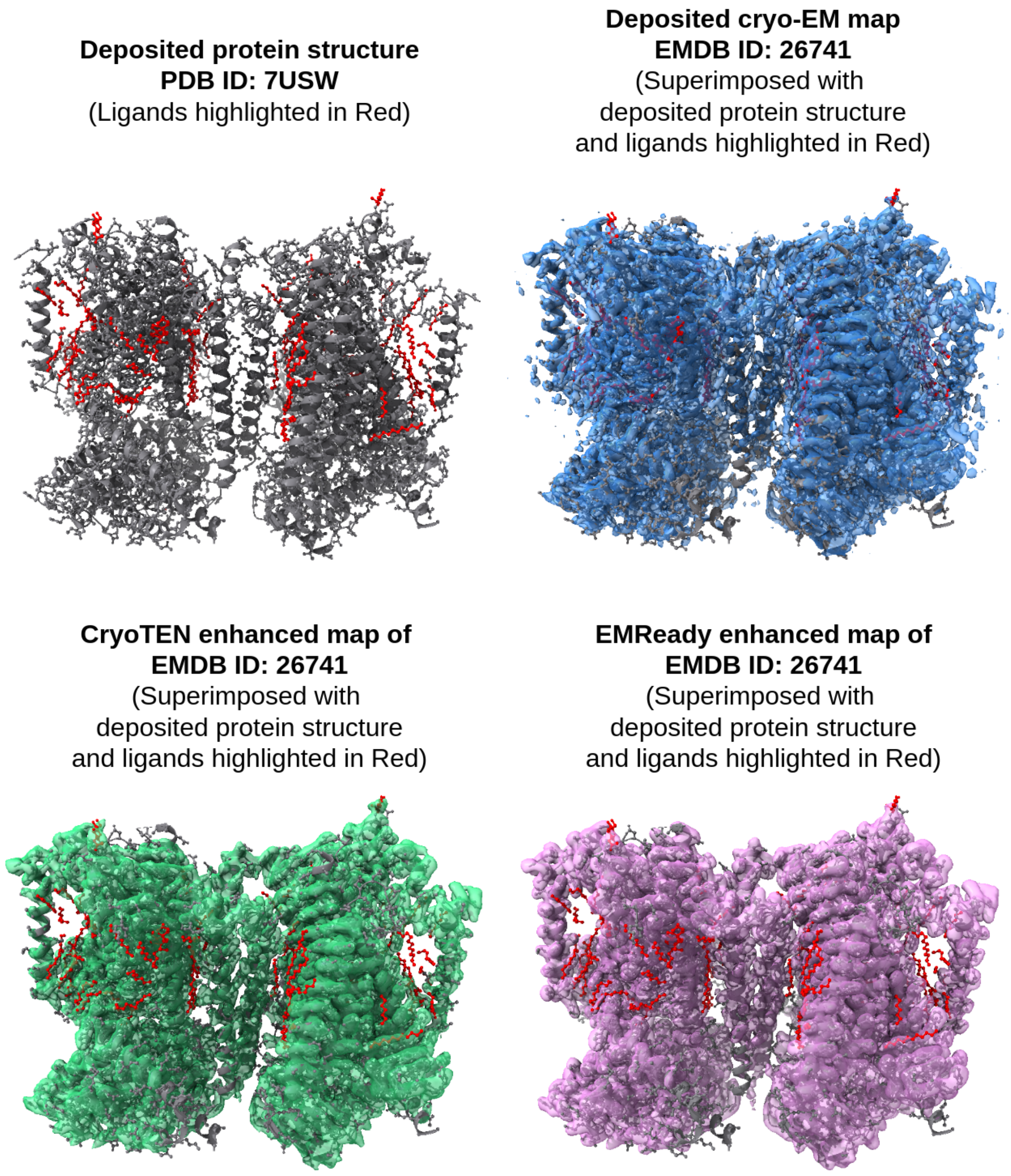

Supplement: btaf092_Supplementary_Data [file btaf092_supplementary_data.pdf]
